# Proanthocyanidins Maintain Cardiac Ionic Homeostasis in Aldosterone-Induced Hypertension and Heart Failure

**DOI:** 10.3390/ijms22179602

**Published:** 2021-09-04

**Authors:** Natalia de las Heras, Adrián Galiana, Sandra Ballesteros, Elena Olivares-Álvaro, Peter J. Fuller, Vicente Lahera, Beatriz Martín-Fernández

**Affiliations:** 1 Department of Physiology, Faculty of Medicine, Plaza Ramón y Cajal, s/n. Universidad Complutense, 28040 Madrid, Spain; nlashera@ucm.es (N.d.l.H.); adrian.galiana@unir.net (A.G.); sballest@ucm.es (S.B.); elenaolivares@ucm.es (E.O.-Á.); vlahera@ucm.es (V.L.); 2 Centre for Endocrinology and Metabolism, Hudson Institute of Medical Research, Clayton, VIC 3168, Australia; peter.fuller@hudson.org.au; 3 Department of Molecular Biology, Faculty of Biology, Universidad de León, Campus de Vegazana s/n, 24071 León, Spain

**Keywords:** hypertension, cardiac hypertrophy, proanthocyanidins, ion homeostasis

## Abstract

Excess aldosterone promotes pathological remodeling of the heart and imbalance in cardiac ion homeostasis of sodium, potassium and calcium. Novel treatment with proanthocyanidins in aldosterone-treated rats has resulted in downregulation of cardiac SGK1, the main genomic aldosterone-induced intracellular mediator of ion handling. It therefore follows that proanthocyanidins could be modulating cardiac ion homeostasis in aldosterone-treated rats. Male Wistar rats received aldosterone (1 mg kg^−1^ day^−1^) +1% NaCl for three weeks. Half of the animals in each group were simultaneously treated with the proanthocyanidins-rich extract (80% *w*/*w*) (PRO80, 5 mg kg^−1^ day^−1^). PRO80 prevented cardiac hypertrophy and decreased calcium content. Expression of ion channels (ROMK, NHE1, NKA and NCX1) and calcium transient mediators (CAV1.2, pCaMKII and oxCaMKII) were reduced by PRO80 treatment in aldosterone-treated rats. To conclude, our data indicate that PRO80 may offer an alternative treatment to conventional MR-blockade in the prevention of aldosterone-induced cardiac pathology.

## 1. Introduction

Primary aldosteronism is a clinical syndrome characterized by hypertension, suppressed plasma renin and autonomous aldosterone production [1,2]. Chronic and severe hypertension leads to cardiac hypertrophy and, eventually, heart failure [3,4,5]. The development of cardiac hypertrophy is frequently accompanied by an imbalance in sodium, potassium and calcium homeostasis leading to alterations in ion currents thereby contributing to development of ventricular dysfunction and arrhythmias [6,7]. Such imbalance in electrolyte homeostasis is, at least in part, a consequence of changes in the expression and activity of key proteins involved in cardiomyocyte ion transport [8,9]. Excess aldosterone promotes pathological remodeling of the heart and an imbalance in cardiac ion homeostasis leading to the development of cardiac hypertrophy, heart failure and arrhythmias [10,11,12,13]. Serum- and glucocorticoid-regulated kinase 1 (SGK1), one of the key mediators of aldosterone action, modulates several ion transporters in the heart [14,15,16,17]. Among these, the sodium–hydrogen exchanger type 1 (NHE1), the Na^+^/K^+^ ATPase pump (NKA) and the sodium-calcium exchanger type 1 (NCX1) play central roles in sodium homeostasis. The NKA resides in both external sarcolemma and T-tubular membrane in cardiac myocytes and plays a fundamental role in the regulation of sodium and potassium concentrations in the final phase of the contraction–relaxation cycle, thus contributing to the return to the resting potential [18]. NHE1 is a transmembrane protein involved in sodium homeostasis, regulation of pH and cell volume through sodium–hydrogen exchange [19,20]. The NHE is one such secondary active transporter, regulating intracellular pH, Na^+^ concentration, and cell volume. NHE1, the major isoform found in the heart, is activated in response to a variety of stimuli including hormones and mechanical stress [21]. NCX1 is crucial for calcium regulation in the cardiomyocyte [6] and its overexpression affects calcium currents, which contributes to the development of diastolic dysfunction and an increased risk of arrhythmia [6]. In certain situations, NCX1 works in the reverse mode depending on the membrane potential and the intracellular concentrations of sodium and calcium. High levels of intracellular calcium favor the normal NCX1 mode (calcium release and sodium entry), while high intracellular sodium drives the NCX1 reverse mode (sodium release and calcium entry) [22]. The renal outer medullary potassium channel (ROMK) is an inwardly rectifying potassium channel member 1 or Kir1.1 ATP-dependent where overexpression has been linked to the development of hypertension [23], while loss of function polymorphisms or pharmacological inhibition protects against hypertension [24,25]. Aldosterone excess, acting via SGK-1also stimulates the expression of the α1C subunit of the L-type voltage-dependent calcium channel (Cav1.2) [10,12]. Furthermore, aldosterone is among the factors that alter the activity of CaMKII by oxidation, which in turn promotes the overactivation of the kinase, resulting in increased apoptosis, arrhythmias and cardiac dysfunction [26,27]. 

Heart failure is a multifactorial pathology and, although, there have been several improvements in treatment favoring survival over last 30 years, the prognosis remains unsatisfactory [28]. In previous studies, we established an exciting line of research into proanthocyanidins and their promising role in cardiovascular treatment through mineralocorticoid receptor (MR) blockade. Treatment with proanthocyanidins preserved cardiac function, reduced fibrosis and inflammation and maintained oxidation/antioxidant homeostasis in aldosterone-induced heart failure [15,29]. In addition, we observed that proanthocyanidins blocked aldosterone-induced cardiac SGK-1 mediated, epithelial sodium channel gamma (γENaC) protein expression and neural precursor cell expressed developmentally down-regulated 4-2 (Nedd4-2) phosphorylation. The observed effects might be the result of blockade of the MR [15,29]. Therefore, we sought to identify the specific mechanisms through which proanthocyanidins are regulating cardiac ion transient in a model of -aldosterone-induced heart failure by measuring NHE1, NKA, NCX1, ROMK, Cav1.2 and CAMKII protein expression.

## 2. Results

### 2.1. Hemodynamics, Cardiac Hypertrophy and Calcium Content

As shown in Table 1, SBP, SBP, DBP, LVEDP and LVSP were higher in the ALDO group than in control. Treatment with PRO80 significantly decreased the parameters increased (*p* < 0.05) by aldosterone-salt administration. Heart rate was comparable in all groups.

Aldosterone administration increased the HW/BW ratio (*p* < 0.05) in the ALDO group compared with control, while PRO80 prevented it (*p* < 0.05) (Table 1).

**Figure 1:**
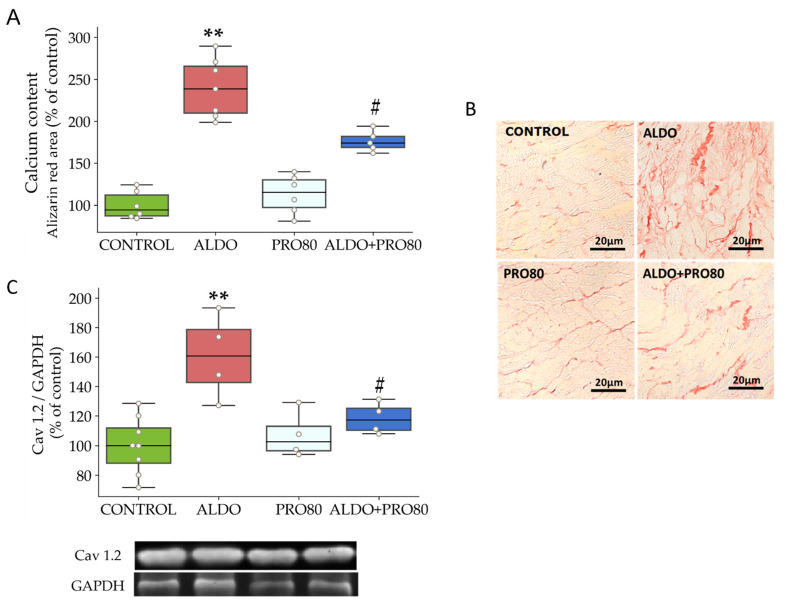
PRO80 effects on aldosterone-induced calcium deposits and myocardial protein expression Cav 1.2. (**A**): Relative quantification of calcium content by alizarin red stained area measurement of myocardial sections. (**B**): Representative photomicrographs of myocardial alizarin red stained sections. (**C**): Relative protein expression levels by western blot of α1C subunit of the L-type voltage-dependent calcium channel (Cav1.2). The boxplots show median, first and third quartile, and minimum and maximum values; the dots show individual values. ** *p* < 0.01 vs. control; # *p* < 0.05 vs. ALDO. control: Control group; ALDO: Aldosterone-salt group; PRO80: Proanthocyanidins-80% enriched extract group; ALDO + PRO80: Aldosterone plus proanthocyanidins 80% enriched extract group (*n* = 8).

Calcium content increased in rats given aldosterone (*p* < 0.01) compared to control rats. The ALDO + PRO80 group showed lower levels of cardiac calcium content than the ALDO group. Figure 1 shows representative micrographs of histological sections of hearts stained with alizarin red in the four experimental groups. In the ALDO group, an increase in the cardiac calcium content, represented by higher orange intensity, was observed compared to the control group. In the ALDO + PRO80 group, a decrease in calcium content can be appreciated in the microphotography when compared to the ALDO group (Figure 1A,B).

Cardiac protein expression of the α1C subunit of the Cav1.2 channel increased in the rats with aldosterone (*p* < 0.01) compared to control rats and decreased in the ALDO + PRO80 group (*p* < 0.01) with respect to the ALDO group (Figure 1C).

### 2.2. Cardiac Protein Expression in Channels

#### 2.2.1. ROMK Protein Expression

**Figure 2:**
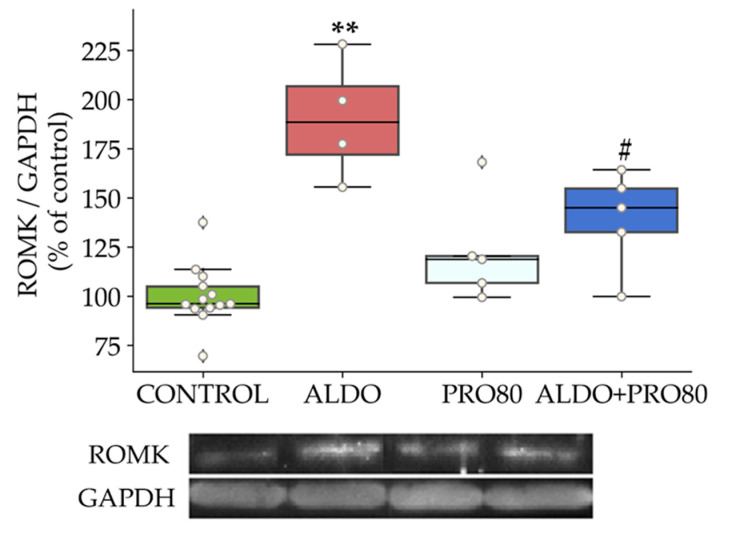
PRO80 effects on aldosterone-disbalanced potassium outflow. Relative protein expression levels of the renal outer medullary potassium channel (ROMK) by western blot. The boxplots show median, first and third quartile, and minimum and maximum values; the dots show individual values. ** *p* < 0.01 vs. control; # *p* < 0.05 vs. ALDO. control: Control group; ALDO: Aldosterone-salt group; PRO80: Proanthocyanidins-80% enriched extract group; PRO80+ALDO: Aldosterone plus proanthocyanidins-80% enriched extract group (*n* = 8).

ROMK levels were higher (*p* < 0.01) in the ALDO group compared with those of control rats, which decreased in the ALDO + PRO80 group (*p* < 0.05) compared to the ALDO group (Figure 2).

#### 2.2.2. Sodium Exchangers: NHE1, NKA and NCX1

**Figure 3:**
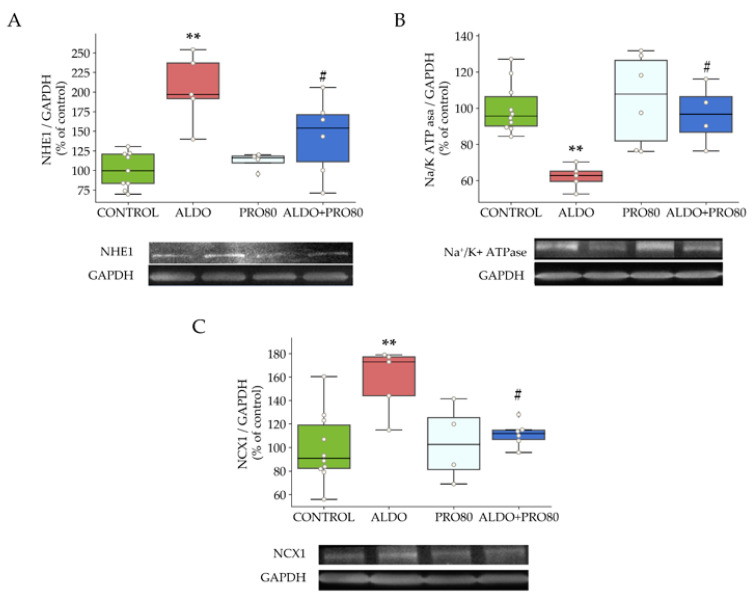
Effect of aldosterone-induced massive entry of sodium; role in NHE1, NKA and NCX1 expression. Relative protein expression levels by western blot of: (**A**): The sodium–hydrogen exchanger type 1 (NHE1), (**B**): The Na^+^/K^+^ ATPase pump (NKA) and (**C**): The sodium-calcium exchanger type 1 (NCX1). The boxplots show median, first and third quartile, and minimum and maximum values; the dots show individual values. ** *p* < 0.01 vs. control; # *p* < 0.05 vs. ALDO. CONTROL: Control group; ALDO: Aldosterone-salt group; PRO80: Proanthocyanidins-80% enriched extract group; PRO80+ALDO: Aldosterone plus proanthocyanidins-80% enriched extract group (*n* = 8).

There was an increase in NHE1 heart protein expression produced by the administration of aldosterone (*p* < 0.01) relative to the control group, which was reversed in the ALDO + PRO80 group (*p* < 0.01) compared to the ALDO group (Figure 3A).

Protein expression of the NKA-α1 subunit decreased (*p* < 0.01) in the ALDO group with respect to the control group; this change was blocked in the ALDO + PRO80 group (*p* < 0.05) compared to ALDO alone (Figure 3B).

Aldosterone administration increased the protein expression of the NCX1 compared to control animals (*p* < 0.01); this was reversed in the ALDO + PRO80 group (Figure 3C).

#### 2.2.3. Calcium Transient Mediators: Total, Phosphorylated and Oxidized CAMKII

**Figure 4:**
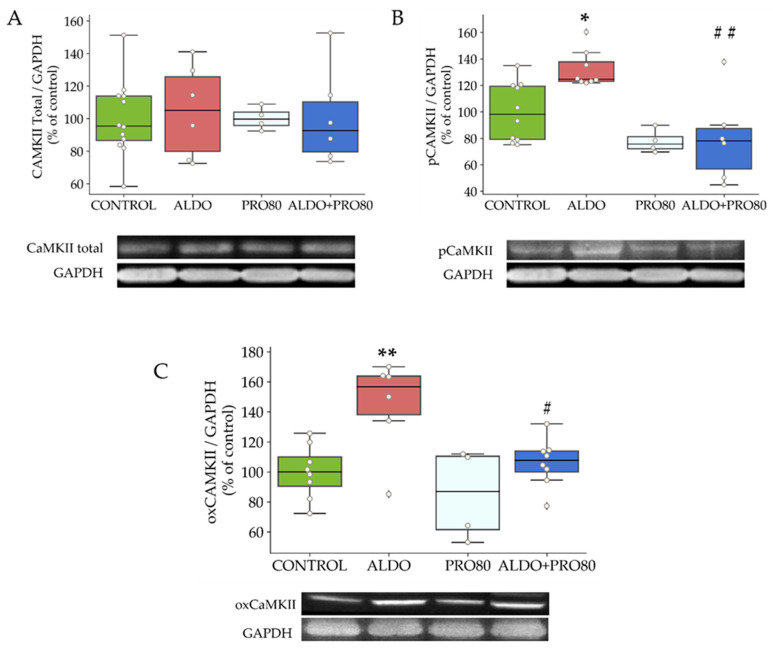
Effect of aldosterone and PRO80 treatment on myocardial protein expression of CAMKII, pCAMKII and oxCAMKII. Relative protein expression levels of Ca^2+^/calmodulin-dependent protein kinase II by western blot. (**A**): Total CaMKII, (**B**): Phosphorylation in threonine 286 (pCaMKII), (**C**): Oxidation in methionines 281 and 282 (oxCAMKII). The boxplots show median, first and third quartile, and minimum and maximum values; the dots show individual values.* *p* < 0.05 vs. control; ** *p* < 0.01 vs. control; # *p* < 0.05 vs. ALDO; ## *p* < 0.01 vs. ALDO. CONTROL: Control group; ALDO: Aldosterone-salt group; PRO80: Proanthocyanidins-80% enriched extract group; PRO80+ALDO: Aldosterone plus proanthocyanidins-80% enriched extract group (*n* = 8).

The levels of total CaMKII did not vary between groups (Figure 4A). However, the level of phosphorylation at threonine 286 (pCaMKII) was increased in rats given aldosterone (*p* < 0.05) relative to control rats. This parameter decreased in the ALDO + PRO80 group (*p* < 0.05) compared to the ALDO group (Figure 4B). On the other hand, levels of cardiac CaMKII oxidation in methionines 281 and 282 (oxCaMKII) increased (*p* < 0.05) in the ALDO group compared to the control group and decreased in the ALDO + PRO80 group (*p* < 0.01) (Figure 4C).

#### 2.2.4. Correlations between Cardiac Function Parameters and Ionic Exchangers Proteins

**Figure 5:**
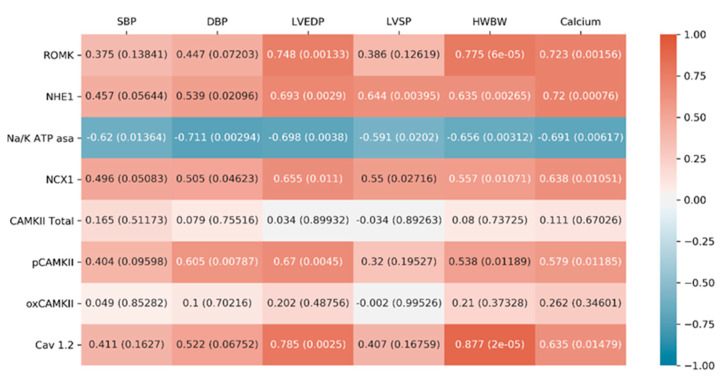
Correlations between cardiac function parameters and ionic exchangers proteins heat map. Systolic blood pressure (SBP), diastolic blood pressure (DBP), left ventricular end diastolic pressure (LVEDP), left ventricular systolic pressure (LVSP), body weight (BW), heart weight (HW), renal outer medullary potassium channel (ROMK), sodium–hydrogen exchanger type 1 (NHE1), Na^+^/K^+^ ATPase pump (NKA), sodium-calcium exchanger type 1 (NCX1), calcium/calmodulin-dependent protein kinase II (CaMKII), phosphorylation in threonine 286 CAMKII (pCaMKII), oxidation in methionines 281 and 282 CAMKII (oxCAMKII) and L-type voltage-dependent calcium channel (Cav1.2).

The correlations results, displayed in Figure 5, are discussed below in the “Discussion” section.

## 3. Discussion

The study provides new findings related to aldosterone-induced cardiac pathology and proanthocyanidins treatment. We have demonstrated, for the first time, aldosterone-mediated regulation of ROMK in the heart. In addition, we show that PRO80 blocked the aldosterone-induced changes in cardiac ROMK, NCX1, NKA (α1 subunit), NHE1, Cav1.2 (α1C subunit), pCAMKII, oxCamKII and protein expression and cardiac calcium content.

### 3.1. PRO80 Maintains potassium Outflow Regulating ROMK Expression

Previous studies have confirmed that aldosterone induces ROMK expression in the kidney [30,31] and that SGK1 stimulates its activity by specific phosphorylation [32,33,34]. Our results show for the first time that aldosterone increases the expression of ROMK in the heart. Co-treatment with PRO80 blocked the increase in expression of ROMK seen with aldosterone alone. Previous studies have shown the beneficial effects of decreased cardiac Kir channel expression levels and/or activity, resulting in improved function and reduced risk of arrhythmias [35,36,37]. The decrease in ROMK expression in the ALDO + PRO80 group compared to the ALDO group could contribute to the maintenance of the potassium outflow during repolarization. Supporting the results, a strong and significant correlation was observed between ROMK and LVEDP, HW/BW and calcium content (Figure 5). Therefore, the data suggest that treatment with PRO80 blocks the ALDO-induced changes in cardiac function and cardiac hypertrophy through ROMK regulation.

### 3.2. PRO80 Prevents Massive Entry of Sodium; Role in NHE1, NKA and NCX1 Expression

As described, the cardiac function of the ALDO group was affected and blocked in the ALDO + PRO80 group. The myocardium is highly sensitive to changes in sodium concentrations inside and outside the cell with increased sodium uptake promoting diastolic dysfunction and the transition from hypertrophy to heart failure [38,39]. To assess cardiac sodium homeostasis, we measured NHE1, NKA and NCX1 protein expression. Aldosterone stimulates NHE1 [40,41] via SGK1-dependent phosphorylation [42,43], resulting in massive entry of sodium into cardiac cells. NHE1 activation results in elevation of pH and intracellular Na^+^ concentration, which potentially enhances downstream signaling cascades in the myocardium and is relevant to hypertension [21]. SGK1 also triggers a cascade that leads to the absorption of sodium through the epithelial Na^+^ channel [44] (ENaC). The activity of this channel is regulated by aldosterone and hormones involved in the maintenance of sodium balance, blood volume and blood pressure. Hence, changes in ENaC activity and/or expression result in cardiac function alteration [15,44]. The levels of cardiac NHE1 were significantly lower in the ALDO + PRO80 group com-pared to the ALDO group. Our results suggest that proanthocyanidins prevent sodium entry through aldosterone induced NHE1, contributing to the restoration of sodium homeostasis and blood pressure regulation as observed by decreased SBP and DBP. Previous studies have shown the effect of certain polyphenols to decrease systolic pressure and confer cardiovascular protection [30]. In our study, NHE1 positively correlated with SBP and DPB supporting the role of proanthocyanidins as a strong antihypertensive agent. Additionally, the observed effect could be directly related to the lower HW/BW ratio observed in the ALDO + PRO80 group compared to the ALDO group as supported by the positive correlation between NHE1 and HW/BW and calcium content and subsequent cardiac function improvement

On the other hand, the NKA alpha subunit decreased in the ALDO group when compared to the control; this effect was blocked by PRO80. Previous studies have reported that aldosterone stimulates NKA activity and/or expression in the heart [45,46]. This apparent discrepancy may reflect differences in the animal models used, measurement of gene expression and/or activity rather than the levels of the alpha subunit, chronic (three weeks) versus 6 h of aldosterone treatment (Ikeda et al.) or the use of adrenalectomized animals (Marver et al). NKA activity and/or expression has been shown to protect against increased sodium entry in cardiomyocytes from animals with heart failure [47,48]. Previous studies have shown that decreased expression and activity of the cardiac NKA pump is associated with production of reactive oxygen species (ROS) [49,50]. Therefore, it is proposed that the increased NKA protein expression in the ALDO + PRO80 group is related to the inhibitory effect of PRO80 on protein expression of cardiac pro-oxidative molecules, NADPH oxidase or inducible nitric oxide synthase (iNOS), as previously described [15]. Zhao et al. have described increased expression of the NKA α1 subunit in isolated rat hearts in an ischemia/reperfusion model when treated with a grape seed proanthocyanidins extract [51]. This is consistent with our observation that aldosterone treatment decreased the subunit protein expression which was reversed by proanthocyanidins co-treatment. Proanthocyanidins could decrease ROS production stimulated by aldosterone in cardiac cells, protecting NKA from oxidation and subsequent degradation. 

The expression of NCX1 was increased in the ALDO group compared to control group. NCX1 works in reverse mode depending on the membrane potential and the intracellular concentrations of sodium and calcium. Slight changes in NCX1 activity significantly alter calcium homeostasis [6,52]. Therefore, the loss of sodium homeostasis induced by NHE1 and NKA dysregulation would activate the reverse mode of NCX1, contributing to compensating the NKA downregulation to expel intracellular sodium excess. A number of studies carried out in animal models of ischemia-reperfusion damage, cardiac hypertrophy and heart failure have shown that lower reverse-activity of NCX1 is associated with an improvement in diastolic function through prevention of intracellular calcium overload [53,54,55,56,57]. Treatment with proanthocyanidins significantly decreased cardiac NCX1 expression in the ALDO + PRO80 group compared to the ALDO group. There was a positive correlation between NCX1 and calcium content supporting the effect of proanthocyanins in decreasing calcium accumulation and cardiac hypertrophy. The effect could be a consequence of NKA restored expression in the ALDO + PRO80 group compared to aldosterone rats.

### 3.3. PRO80 Controls Calcium Homeostasis by Regulating Cav1.2 and CAMKII Expression

Excessive calcium entry in cardiac cells promotes pathological remodeling resulting in cardiac hypertrophy. Aldosterone increases calcium influx currents in cardiomyocytes through Cav1.2 overexpression, leading to cardiac hypertrophy and diastolic dysfunction [12,58]. Cardiac protein expression of α1C Cav1.2 increased significantly in the ALDO group compared to the control group.

Our results show for the first time that proanthocyanidins reduce aldosterone-induced autophosphorylation of cardiac CaMKII. In the ALDO + PRO80 group, treatment with proanthocyanidins was able to significantly reduce pCaMKII cardiac levels. The reduced amount of cardiac calcium content observed in the ALDO + PRO80 group compared to the ALDO group would reduce the number of activated and bound calcium-calmodulins to CaMKII. As a result, autophosphorylation of the CaMKII would be reduced in ALDO + PRO80 rats. Certain polyphenols have shown the ability to reduce CaMKII autophosphorylation and oxidation [59,60]. The results suggest that proanthocyanidins could prevent, at least in part, CaMKII overactivation by lowering intracellular calcium overload and ROS production in aldosterone-treated rats. The effect would contribute to the lowering of the HW/BW ratio and subsequent reduced cardiac hypertrophy through modulation of activation and expression of Ca^2+^ cycling proteins.

Cardiac protein expression of α1C Cav1.2 increased in ALDO group compared to control group L-type Ca^2+^ channels are regulated by a change in membrane potential [61]. Their activity is modulated further by hormones such as aldosterone resulting in calcium overload in cardiomyocytes which is highly involved in cardiac hypertrophy development and subsequent alteration of cardiac function. Low-voltage-activated (LVA) T-type calcium channel overexpression also contributes to cardiac hypertrophy when overexpressed [62,63]. These channels are involved in the cardiac pacemaker function and if overexpressed in myocytes confer contractile automaticity to the cells. On the other hand, cardiac protein expression of α1C Cav1.2 decreased significantly in the ALDO + PRO80 group compared to the ALDO group. Seminal studies have shown that MR antagonists prevent aldosterone-induced upregulation of Cav1.2, enabling restoration of the calcium current [58,64]. As we previously proposed, proanthocyanidins seem to act as MR antagonists. In addition, Cav1.2 blockade has shown beneficial effects in animal models of hypertension by preventing intracellular calcium overload in cardiomyocytes, resulting in less cardiac remodeling, cardiac hypertrophy and diastolic dysfunction [65]. The decreased expression of cardiac NCX1 together with that of Cav1.2 observed in the ALDO + PRO80 group with respect to ALDO group would contribute to the re-establishment of calcium homeostasis. These results would be related to the lower HW/BW ratio showed in the ALDO + PRO80 group compared to the ALDO group, which would mean reduced cardiac hypertrophy.

## 4. Materials and Methods

### 4.1. Experimental Design and Animal Model

The study was conducted on 32 male Wistar rats (250 g; Harlan Ibérica, Barcelona, Spain). The Universidad Complutense Ethics Review Board specifically approved this study according to the guidelines for ethical care of experimental animals of the European Union following the National Guideline 53/2013. Rats were fed standard rat chow and tap water ad libitum and kept in a quiet room at constant temperature (20–22 °C) and humidity (50–60%). Before allocating animals to treatment, blood pressure was measured to group them under the same mean systolic blood pressure by the tail-cuff method. Then, rats were divided into four groups (*n* = 8 per group): ALDO—subcutaneous aldosterone injection (1.5 mg kg^−1^ day^−1^, Sigma Aldrich, Germany) once daily dissolved in vehicle (sunflower oil) plus 1% NaCl as drinking water; PRO80—proanthocyanidins-rich extract (80% *w*/*w*, Natac Biotech, Madrid, Spain) dissolved in drinking water (5 mg kg^−1^ day^−1^); ALDO + PRO80—rats injected with aldosterone plus 1% NaCl as drinking water and treated with PRO80 dissolved in drinking water (5 mg kg^−1^ day^−1^); and control rats (control). The aldosterone plasma concentration was analyzed in a previous study using a specific quantitative sandwich enzyme immunoassay (Cayman Chemical, Cayman, MI, USA); it was significantly increased in Aldo-salt-treated animals compared with control animals (700 ± 144 vs. 275 ± 42 pg/mL, *p* < 0.01) [12]. PRO80 consisted of a dry cranberry extract with 80% proanthocyanidins in its composition (Table S2). Control and PRO80 animals were also subcutaneously injected with vehicle once daily. The treatment period was three weeks. Then, rats were sacrificed and the heart weight to body weight ratio (HW/BW) was calculated as an index of cardiac hypertrophy.

### 4.2. Hemodynamics

Before sacrifice, animals were anesthetized (Ketamine, Imalgene 1000, 70 mg/Kg, and Diazepam 1 mL/kg: intraperitoneal injection). A catheter (Science FT211B, 1.5-mm diameter) was inserted into the right carotid artery where systolic blood pressure (SBP) and diastolic blood pressure (DBP) were measured (*n* = 10 per rat). Next, the catheter was advanced into the left ventricle and the left ventricular function was measured as previously described [12,15]. Heart rate (HR) left ventricle end diastolic pressure (LVEDP) and left ventricle systolic pressure (LVSP) were measured (*n* = 10 per rat). The catheter was connected to a data-acquisition system (PowerLab/800, AD Instruments), and signals were monitored and digitally stored for analysis with Chart for Windows software. The measurements were obtained by a researcher unaware of the treatments. 

### 4.3. Histological Analysis

To assess calcium content, paraffin-embedded heart 4-mm slices were stained with alizarin red (acid; Aldrich Chemical Company, Madrid, Spain). Four different sections of each slide of the heart and ten photographs from each section were taken using an image analysis system (Leica Microsystems, Barcelona, Spain). A single investigator, blinded to the nature of the samples, performed the analyses.

### 4.4. Western Inmunoblotting

Frozen heart samples (100 mg) were homogenized using an automatic homogenizer (Bullet blender, Next-Advance, USA) on ice-cold lysis buffer, containing 150 mM Tris pH 7.4, 50 mM NaCl, 1% Triton X-100, 3 mM phenylmethylsulfonylfluoride, 3 mM dithiothreitol (Sigma, Spain) and one tablet of protease inhibitor cocktail (Roche, Mannheim, Germany). Homogenates were centrifuged at 13,000 rpm at 4 °C for 15 min. Purified proteins from the supernatant were collected and stored at −80 °C. For western blot analysis, Laemmli buffer (Bio-Rad, Hercules, CA, USA) was added. Then, the samples were boiled for five minutes at 95 °C and placed on ice for 10 min before electrophoresis. Proteins were separated on SDS-PAGE gels and transferred to nitrocellulose membranes (Bio-Rad, USA). The membranes were blocked for 1 h with 5% (*w*/*v*) BSA as blocking agent (Sigma, Madrid, Spain) in PBST (1% PBS, 0.1%Tween 20 *v*/*v*) at room temperature. After washing with PBST, the membranes were probed overnight at 4 °C with appropriate primary antibodies, collected in Table 2. After washing, the membrane was incubated for 1 h with peroxidase-conjugated rabbit or mouse anti-goat IgG secondary antibody (1:10,000). For detection, ECL Advance Western Blotting Detection kit (Amersham, UK) was used. Blots were probed with rabbit monoclonal anti-GAPDH antibody (1:10,000, Abcam, Cambridge, UK) as internal control, to normalize between gels. Quantification was expressed as percentage of relative protein expression (Protein/GAPDH) vs. control group.

### 4.5. Statistical Analysis

In this experiment, we used python 3.7.6 language libraries for data processing. Statistical tests of normality (Shapiro-Wilk) and significance (one-way ANOVA) for the studied variables were performed. Tukey’s test was used for post hoc analyses. Pearson correlation coefficients were used to examine the association among different variables. The data were represented in boxplots showing median, first and third quartile, and minimum and maximum values; the dots show individual values. The level of significance was set at *p* < 0.05. Table S1 compiles the number of analyzed and excluded animals.

## 5. Conclusions

**Figure 6:**
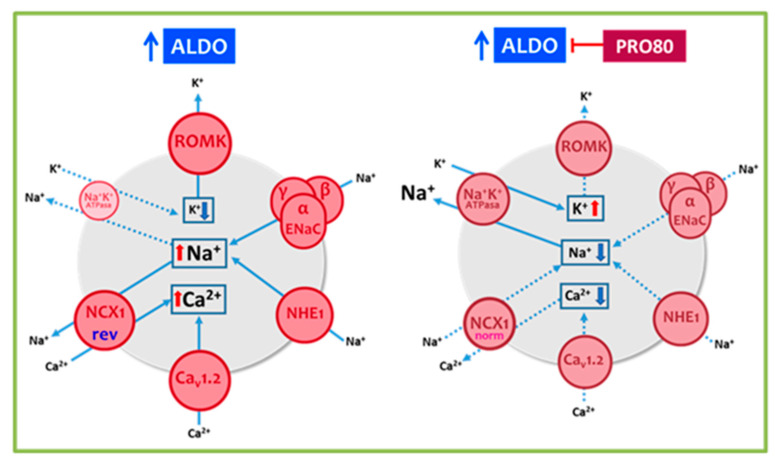
Scheme of the proposed actions of aldosterone and proanthocyanidins in cardiac sodium, potassium and calcium homeostasis by modifying protein expression of the ion channels. Sodium–hydrogen exchanger type 1; NHE1, Na^+^/K^+^ ATPase pump; NKA, sodium-calcium exchanger type 1; NCX1, renal outer medullary potassium channel; ROMK, L-type volt-age-dependent calcium channel; Cav1.2, calcium/calmodulin-dependent protein kinase II; CaMKII and epithelial sodium channel (ENaC). Full lines represent increased ion transient and dotted lines represent decreased ion transient trough the transporters.

The proanthocyanidins-based treatment, PRO80, prevented aldosterone-induced changes in the cardiac protein expression of ion transporters (ROMK, NHE1, NKA, Cav1.2 and NCX1), cardiac calcium content and CaMKII autophosphorylation and oxidation levels. Figure 6 summarizes the proposed actions of aldosterone and proanthocyanidins in cardiac sodium, potassium and calcium homeostasis by modifying protein expression of the ion channels. Through these mechanisms, PRO80 may contribute to reversing the alterations in ion balance in conditions of aldosterone excess, thereby reducing cardiac hypertrophy and improving cardiac function. These results support and expand the evidence, which point to PRO80 as an alternative treatment to prevent aldosterone-induced cardiac alteration pathologies.

## Figures and Tables

**Table 1:** Hemodynamic parameters, body weight and relative heart weight.

	CONTROL	ALDO	PRO80	ALDO + PRO80
SBP (mmHg)	108 ± 0.9	144 ± 4.3 *	115 ± 2.6	128 ± 1.1 ^#^
DBP (mmHg)	76 ± 5.1	116 ±3.7 *	71 ± 4.2	88 ± 2.9 ^#^
LVSP (mmHg)	113 ± 3.1	147 ± 5.1 *	124 ± 7.1	124 ± 7.8 ^#^
LVEDP (mmHg)	3.8 ± 0.3	13 ± 0.9 *	4.6 ± 0.2	4.5 ± 0.3 ^#^
BW baseline (g)	253 ± 2.5	248 ± 6.2	261 ± 7.5	246 ± 3.2
BW final (g)	323 ± 3.1	315 ± 4.3	330 ± 6.6	318 ± 5.2
HW/BW (mg g^−1^)	2.28 ± 0.06	3.20 ± 0.1 *	2.32 ± 0.06	2.43 ± 0.08 ^#^

Abbreviations: SBP, systolic blood pressure; DBP, diastolic blood pressure; LVSP, left ventricular systolic pressure; LVEDP, left ventricular end diastolic pressure; BW, body weight; HW, heart weight. * *p* < 0.05, versus *CT*; ^#^ *p* < 0.05, versus ALDO.

**Table 2:** Antibodies, commercial references (ab: Abcam; sc: Santa cruz; mp: Millipore; c-cst: Sigma) and conditions used concerning the proteins studied by western blot.

Antibody	Ref.	Dilution
anti-ROMK	ab92285	1:250
anti-NHE1	ab67314	1:500
anti-1α NKA	ab7671	1:1000
anti-α1C Cav1.2	ab58552	1:500
anti-NCX1	ab6495	1:500
anti-CaMKII	ab52476	1:500
anti-pCaMKII	ab32678	1:500
anti-oxCaMKII	mp07-1387	1:500

## Data Availability

The data presented in this study are available upon request from the corresponding authors.

## Supplementary Materials

The following are available online at https://www.mdpi.com/article/10.3390/ijms22179602/s1.
