# Supplementary material for: Proanthocyanidins Maintain Cardiac Ionic Homeostasis in Aldosterone-Induced Hypertension and Heart Failure
Source: Int J Mol Sci. 2021 Sep 4;22(17):9602. doi: 10.3390/ijms22179602 (PMC8431754; doi:10.3390/ijms22179602)
Supplement: Supplementary file 1 [file ijms-22-09602-s001.zip › Table S2.pdf]

Table S2. PRO80 full composition.

	PRO80 Composition (%)
**Proanthocyanidins**	80
**Catechins**	6.4
**Epicatechins**	4.3
**Anthocyanins**	3.2
**Benzoic acids**	2.7
**Cinnamic acids**	1.8
**Maltodextrin**	0.9
**Pectines**	0.7
